# The investigation of fermented food consumption on gastrointestinal symptoms: a cross-sectional study in university students

**DOI:** 10.7717/peerj.20479

**Published:** 2025-12-16

**Authors:** Hande Seven Avuk, Oyku Aydin, Neslihan Kocatepe, Sude Melis Sahin, Irem Akdogan, Eser Cavus

**Affiliations:** 1 Department of Nutrition and Dietetics, Faculty of Health Sciences, Istanbul Bilgi University, Istanbul, Turkey; 2 Department of Nutrition and Dietetics, Faculty of Health Sciences, Istanbul Kent University, Istanbul, Turkey

**Keywords:** Defecation, Eating habits, Fermented foods, Gastrointestinal tract

## Abstract

**Background:**

Fermented foods (FFs) are a valuable source of live microorganisms that can enhance human health, particularly gastrointestinal health, akin to probiotics. This study investigated the relationship between FFs consumption habits and gastrointestinal system findings in university students.

**Methods:**

A cross-sectional anonymous survey included 546 university students, collecting data on sociodemographic characteristics, the Gastrointestinal Symptom Rating Scale (GSRS), and the Bristol Stool Form Scale (BSFS). FFs consumption habits were recorded using a food frequency questionnaire.

**Results:**

Results showed that the average GSRS score was 33.66 ± 16.89. According to the BSFS, 15% of students experienced constipation, 72.7% had moderate stool consistency, and 12.3% had diarrhea. Notably, kefir, vinegar, and sourdough bread were never consumed by 56.8%, 44.3%, and 41.9% of students, respectively. Significant differences were found between the frequency of yogurt, kefir, vinegar, cheese, pickle consumption, and gastrointestinal symptoms (*p* < 0.05). Students with normal stool consistency had fewer dyspepsia, abdominal pain, reflux symptoms, and overall gastrointestinal symptoms than those with constipation (*p* < 0.05).

**Conclusion:**

The study concludes that FFs such as yogurt, cheese, pickles, and kefir may offer health benefits, reducing symptoms of reflux, dyspepsia, abdominal pain, and general gastrointestinal issues in young adults.

## Introduction

Fermented foods (FFs) have been a component of the human diet for approximately 10,000 years (Leeuwendaal et al., 2022). Pottery vessels from 7000 BC in China, used for fermenting honey, rice and fruit provides some of the earliest evidence of the deliberate practice of fermentation (McGovern et al., 2004). Furthermore, although not conclusive, evidence of drinking beer made from wild plants at Göbekli Tepe at the dawn of the Neolithic period suggests that one of the earliest evidence of FFs may have been obtained in present day Turkey (Dietrich et al., 2012). Recently, the growing awareness that individuals can reduce their risk of disease by adopting healthy lifestyles, including diet, has increased interest in natural foods such as FFs (García-Barón et al., 2025). FFs are defined as “foods obtained through desired microbial growth and enzymatic transformations of food components” (Marco et al., 2021). FFs are categorized according to their live microorganism content. For example, some FFs contain live microorganisms (*e.g.*, yogurt, kefir, sour cream, most cheeses, boza), while others do not (*e.g.*, bread, pasteurized fermented vegetables, vinegar) (Marco et al., 2021). Furthermore, FFs can be categorized as dairy products, fermented cereals, vegetables, fruits, legumes or meat and fish-based products (Salas-Millán & Aguayo, 2024). Fermentation has been used for many purposes, such as improving organoleptic properties, extending shelf life and increasing the bioavailability of nutrients (Nkhata et al., 2018). While a vast diversity of FFs are consumed globally, prominent examples in many cultures, including Turkey, are yogurt, cheese, butter, kefir, ayran, vinegar, pickles, sourdough bread, and olives ((Chilton, Burton & Reid, 2015; Cuamatzin-García et al., 2022; Kabak & Dobson, 2011; Kundakci, Aktac & Gunes, 2021). Identifying FFs bioactive peptide and microbial metabolite content has led to the idea that they may also benefit health (Leeuwendaal et al., 2022). The probiotic effects of microorganisms found in foods, bioactive components obtained from fermentation, and increased absorption of nutrients such as vitamins and prebiotics are among the potential health effects of FFs (Shah et al., 2023). FFs may benefit health through immune system modulation, the presence of bioactive components that may affect intestinal and systematic function, or modulation of gut microbiota activity and composition (Marco et al., 2021). Some meta-analyses of cohort studies have reported potential associations between higher consumption of certain fermented foods, particularly yogurt and other dairy products, and a reduced risk of diseases such as diabetes and cardiovascular disease (Zhang, Bai & Deng, 2022; Zhang et al., 2019; Zhang et al., 2020). However, this field of research is still emerging. The evidence for direct preventative effects is considered preliminary, especially as findings from randomized controlled trials remain limited, and a scientific consensus has not yet been reached.

According to recent data, the incidence of gastrointestinal system diseases worldwide in 2019 increased by approximately 74% compared to 1990, reaching an estimated 444 million (Wang et al., 2023). It has been shown that microbiota changes due to factors such as changes in eating habits, obesity, insufficient physical activity, increased urbanization, and alcohol and cigarette use cause symptoms in the gastrointestinal system (Hall & Crowe, 2011; Wang et al., 2023). The gastrointestinal system is responsible for motility, secretion, digestion, absorption, and elimination of waste products from the body, and a healthy gastrointestinal system is essential for human health. Common gastrointestinal symptoms include abdominal pain, bloating, dyspepsia, nausea, vomiting, heartburn, constipation, and diarrhea (Greenwood-Van Meerveld, Johnson & Grundy, 2017). It is known that gastrointestinal symptoms such as chronic diarrhea, functional dyspepsia, and constipation are more common in female individuals (Narayanan, Anderson & Bharucha, 2021).

The recent interest in gastrointestinal health has increased awareness of FFs (Dimidi et al., 2019). A review of studies has shown that various FFs may have positive effects on conditions such as celiac disease, dyspepsia, Helicobacter Pylori infection, functional constipation, lactose malabsorption, irritable bowel syndrome, and gastrointestinal symptoms (bloating, gas, and diarrhea) (Valentino et al., 2024). In a study conducted by Turan et al. (2014), it was observed that consuming kefir for four weeks in individuals with functional constipation contributed to increased bowel frequency, decreased laxative use, and improved stool consistency. In a randomized, double-blind study, it was determined that consuming unpasteurized and pasteurized sauerkraut for six weeks in 34 individuals with irritable bowel syndrome had a positive effect on the quality of life, satisfaction with bowel habits, and frequency of abdominal pain. Interestingly, that study found that while sauerkraut consumption improved IBS symptoms, the positive effects were observed regardless of whether the product was pasteurized, with no significant difference found between the group consuming the unpasteurized product and the group consuming the pasteurized one (Nielsen et al., 2018). Considering the variability of FFs in terms of fermentation processes and microorganisms, there is limited evidence as to whether they affect gastrointestinal health (Dimidi et al., 2019). This study was conducted to examine the effects of traditionally best-known FFs on consumption habits and gastrointestinal symptoms.

## Materials & Methods

### Study design and participants

This cross-sectional study was conducted on university students who volunteered to participate in the research and were over 18 years of age between April and December 2023 in İstanbul. All procedures involving human participants adhered to the ethical standards of the İstanbul Bilgi University on Human Research Ethics Committee, following the 1964 Helsinki Declaration and its subsequent amendments or comparable ethical standards (project number: 20507-017, date: 30.03.2023). The written informed consent was obtained from all individual participants included in the study. The sample size of the study was found to be at least 374 people, consistent with a similar study and with a margin of error of 0.05 and a power of 0.80 with the G*Power 3.1.9.4 power analysis program (Faul et al., 2007; Sousa, Baptista & Silva, 2022). Exclusion criteria were individuals under 18 years of age; pregnant or breastfeeding women; individuals with a diagnosed chronic disease, a known food allergy or intolerance, or regular medication use; those using probiotic or prebiotic supplements; and individuals with special dietary requirements. A total of 546 university student volunteers, with an average age of 21.84 ± 3.77 years, participated in the study and, the data were collected by face-to-face interview.

### Data collection tools

Survey form: Participants’ general information, health information, Gastrointestinal Symptom Rating Scale (GSRS), Bristol Stool Form Scale (BSFS), and food consumption frequency were collected by face-to-face interview.

Antropometric measurement: The participants’ body weight in the study were measured using a digital scale on a flat and hard surface without shoes. The heights of individuals were measured with the help of a height meter in an upright position, with their feet side by side and their heads on the Frankfurt plane, with their heels against the wall (Norton, 2018). Individuals’ body mass index (BMI) (kg/m^2^) was calculated using the equation (Body weight (kg)/Height (m^2^)) using their body weight (kg) and height (m). The BMI value classification of individuals was made according to the World Health Organization classification (World Health Organization, 2004).

Gastrointestinal Symptom Rating Scale: GSRS, developed by Revicki et al. (1998) and validated in Turkish by Turan, Ast & Kaya (2017), was used to assess common symptoms of gastrointestinal disorders. GSRS asks how the individual has felt regarding gastrointestinal problems in the last week. The scale consists of five sub-dimensions, namely diarrhea (three questions), dyspepsia (four questions), constipation (three questions), abdominal pain (three questions) and reflux (two questions), and a total of 15 questions. The higher the score obtained from the scale, the more severe the gastrointestinal symptoms.

Bristol Stool Form Scale: The Bristol Stool Form Scale is a subjective scale developed by Lewis and Heaton in Bristol, England, used to evaluate an individual’s stool consistency and bowel habits. This scale evaluates defecation status in seven groups: Type 1 and Type 2 constipation; type 3, Type 4, and Type 5 normal defecation; and Type 6 and Type 7 diarrhea (Blake, Raker & Whelan, 2016; Lewis & Heaton, 1997). The authors have permission to use this instrument from the copyright holders.

Fermented food consumption: To assess the dietary intake of FFs, a food frequency questionnaire (FFQ) was utilized, a method commonly used to explore relationships between nutrition and disease (Cade et al., 2002). The selection of foods for the questionnaire was based on two primary criteria: their high consumption prevalence in Turkish society and their alignment with the scientific definition of FFs as established by the ISAPP consensus panel (Marco et al., 2021). The final FFQ, developed for this study, included nine products. These were: whole-fat dairy products from cow’s milk (yogurt, cheese, butter, kefir, and ayran); traditionally fermented, unpasteurized vinegar; unpasteurized, lactic acid-fermented pickles; sourdough bread; and lactic acid-fermented olives. For each food item, participants were asked to report their consumption frequency using seven categories: ‘every day’, ‘5–6 times a week’, ‘3–4 times a week’, ‘1–2 times a week’, ‘once every 15 days’, ‘once a month’, and ‘never’.

### Statistical methods

In this study, the IBM SPSS Statistics 28.0 (IBM SPSS Statistics for Windows, Version 28.0. IBM Corp., Armonk, NY, USA) package program was used for the statistical evaluation of the data. The confidence interval of the statistical tests was accepted as 95%, and the significance level was evaluated as *p* < 0.05. Qualitative variables were expressed as number (n) and percentage (%), and quantitative variables were expressed as mean ($\bar {x}$) and standard deviation (SD). The data conformity to normal distribution was checked with the Kolmogorov–Smirnov test. Since the data did not show normal distribution, descriptive statistics were shown with median and minimum-maximum (min-max) values. Kruskal Wallis Test was used to determine whether there was a difference between the variables, and *Post-hoc* analyses were performed with the Tamhane T2 test considering the distribution of variances.

Multiple linear regression was used to estimate the effects of independent variables on the dependent variable GSRS. The independent variable, fermented food consumption frequency, was constructed similarly to Taylor et al. (2020). According to the frequency of fermented food consumption, participants were grouped as “non-consumers” (1 in 15 days, 1 in a month and never consumed) and “consumers” (consumed every day, 5–6 times a week, 3–4 times a week and 1–2 times a week); multiple linear regression analysis was performed based on this classification. Results are reported as standardized beta (β) coefficients with 95% confidence intervals and *p*-values. A *p*-value <0.05 was considered statistically significant.

## Results

Table 1 demonstrates the general characteristics of participants. A totally 546 student participated th study. The mean age of the university students was 21.84 ± 3.77 years, and the mean BMI was 22.12 ± 3.43 kg/m^2^. In this study, where 90.1% of the participants were female, 96.7% of the participants were single, 83.9% were unemployed and, 54% were living with their families. The mean total score of the GSRS was 33.66 ± 16.89, and the mean scores of its sub-factors were dyspepsia 10.98 ± 6.14, abdominal pain 6.95 ± 3.98, constipation 6.23 ± 4.56, and diarrhea symptom 5.32 ± 3.78. According to the BSFS, it was determined that 15% of the participants had constipation, 72.7% were normal, and 12.3% were diarrheal.

**Table 1:** Characteristics of participants.

**Variables**		***n* = 546**	
**Age (years) (Mean** **± SD)**		21.84 ± 3.77	
**Height (cm)**		166.20 ± 8.87	
**Body Weight (kg)**		60.98 ± 9.86	
**BMI (kg/m** ^2^ **)**		22.12 ± 3.43	
		Number (n)	Percentage (%)
**Gender**	Male	54	9.9
	Female	492	90.1
**Smoking**	Yes	138	25.3
	No	408	74.7
**Alcohol use**	Yes	238	43.6
	No	308	56.4
**BSFS**	Constipation	82	15.0
	Normal	397	72.7
	Diarrhea	67	12.3
**GSRS** **(Mean** **± SD)**	Reflux	4.16 ± 3.05	
	Dyspepsia	10.98 ± 6.14	
	Diarrhea	5.32 ± 3.78	
	Constipation	6.23 ± 4.56	
	Abdominal pain	6.95 ± 3.98	
**GSRS- Total (Mean** **± SD)**		33.66 ± 16.89	

**Notes.**

Data are presented as mean ± SD, or n (%); BMI, Body Mass Index; GSRS, Gastrointestinal Symptom Rating Scale; BSFS, Bristol Stool Form Scale.

Table 2 presents the frequency of consumption of fermented products by participants. We found that 16.1% of the participants consumed yogurt, 32.4% cheese, and 26.4% olives daily. According to the frequency of kefir consumption, 56.8% of the individuals never consumed it. According to the frequency of sourdough bread consumption, it was determined that 41.9% of the individuals never consumed it, and 11.9% consumed it 1–2 times a week. According to the frequency of vinegar consumption, it was determined that 44.3% of the individuals never consumed it. It was seen that 30.8% of the individuals consumed ayran, 26.4% yogurt, 23.6% pickles, and 18.1% butter 1–2 times a week.

**Table 2:** Frequency of consumption of fermented products by participants.

**Fermented foods** **consumption frequency**	**Every day**		**5–6 per week**		**3–4 per week**		**1–2 per week**		**Once every 15 days**		**Once a month**		**Never**	
	Number (n)	Percentage (%)	Number (n)	Percentage (%)	Number (n)	Percentage (%)	Number (n)	Percentage (%)	Number (n)	Percentage (%)	Number (n)	Percentage (%)	Number (n)	Percentage (%)
Yoghurt	88	16.1	92	16.8	155	28.4	147	26.9	43	7.9	12	2.2	9	1.6
Kefir	7	1.3	14	2.6	11	2.0	68	12.5	45	8.2	91	16.7	310	56.8
Ayran	13	2.4	36	6.6	91	16.7	168	30.8	132	24.2	76	13.9	30	5.5
Vinegar	17	3.1	18	3.3	41	7.5	76	13.9	71	13.0	81	14.8	242	44.3
Cheese	177	32.4	101	18.5	111	20.3	87	15.9	27	4.9	12	2.2	31	5.7
Butter	96	17.6	75	13.7	129	23.6	99	18.1	52	9.5	42	7.7	53	9.7
Pickles	26	4.8	54	9.9	89	16.3	129	23.6	107	19.6	86	15.8	55	10.1
Sourdough bread	37	6.8	35	6.4	55	10.1	65	11.9	57	10.4	68	12.5	229	41.9
Olive	144	26.4	82	15.0	93	17.0	86	15.8	42	7.7	29	5.3	70	12.8

**Notes.**

Data are presented as n (%).

**Table 3:** GIS symptoms and sub-dimensions according to the frequency of fermented food consumption.

	**Every day**	**5–6 per week**	**3–4 per week**	**1–2 per week**	**Once every** **15 days**	**Once a month**	**Never**	**Overall *p*-value**
**Yogurt**								
Diarrhea	4.00 (3–21)	4.00 (3–21)	4.00 (3–21)	4.00 (3–21)	3.00 (3–21)	3.50 (3–15)	3.00 (3–5)	0.532
Dyspepsia	9.50 (4–28)	9.00 (4–28)	9.00 (4–28)	9.00 (4–28)	8.00 (4–25)	10.50 (4–28)	7.00 (5–16)	0.984
Constipation	4.00 (3–21)	4.00 (3–21)	4.00 (3–21)	4.00 (3–21)	3.00 (3–21)	4.00 (3–20)	5.00 (3–11)	0.896
Abdominal pain	6.00 (3–21)	6.00 (3–20)	6.00 (3–19)	6.00 (3–21)	5.00 (3–15)	6.00 (3–17)	5.00 (3–8)	0.554
Reflux	4.00^a^ (2–14)	3.00 (2–14)	2.00 (2–14)	3.00 (2–14)	2.00^b^ (2–11)	5.00 (2–14)	3.00 (2–14)	0.005
GIS Total	30.00 (15–99)	20.00 (15–94)	29.00 (15–96)	29.00 (15–99)	26.00 (15–91)	31.50 (15–94)	29.00 (18–35)	0.849
**Kefir**								
Diarrhea	3.00 (3–10)	3.00 (3–8)	4.00 (3–6)	3.50 (3–21)	4.00 (3–21)	4.00 (3–21)	4.00 (3–21)	0.632
Dyspepsia	5.00^a^ (4–8)	11.00^b^ (5–20)	6.00^a^ (4–10)	8.00^d^ (4–28)	9.00^d^ (4–23)	10.00^d^ (4–28)	9.00^d^ (4–28)	0.005
Constipation	4.00 (3–7)	4.00 (3–10)	4.00 (3–5)	4.00 (3–20)	4.00 (3–17)	5.00 (3–21)	4.00 (3–21)	0.323
Abdominal pain	4.00 (3–8)	7.50 (4–15)	6.00 (4–8)	5.00 (3–19)	6.00 (3–20)	6.00 (3–21)	6.00 (3–21)	0.312
Reflux	2.00 (2–4)	3.50 (2–14)	3.00 (2–8)	2.50 (2–14)	3.00 (2–12)	3.00 (2–14)	3.00 (2–14)	0.319
GIS -Total	20.00^a^ (15–30)	33.50^b^ (18–49)	24.00^ab^ (18–32)	26.00^c^ (15–84)	27.00^bc^ (15–66)	31.00^d^ (15–99)	30.00^d^ (15–94)	0.038
**Ayran**								
Diarrhea	6.00 (3–20)	5.00 (3–21)	3.00 (3–18)	4.00 (3–21)	4.00 (3–21)	4.00 (3–19)	5.00 (3–18)	0.101
Dyspepsia	8.00 (4–24)	9.00 (4–28)	8.00 (4–28)	9.00 (4–28)	8.50 (4–28)	9.00 (4–28)	13.00 (4–28)	0.380
Constipation	3.00 (3–13)	5.00 (3–21)	4.00 (3–21)	4.00 (3–21)	4.00 (3–21)	4.50 (3–21)	6.50 (3–20)	0.146
Abdominal pain	7.00 (3–18)	6.00 (3–21)	5.00 (3–19)	6.00 (3–21)	6.00 (3–19)	5.00 (3–21)	6.00 (3–17)	0.507
Reflux	3.00 (2–14)	4.00 (2–14)	3.00 (2–14)	3.00 (2–14)	3.00 (2–14)	3.00 (2–14)	4.00 (2–14)	0.371
GIS -Total	32.00 (16–84)	31.50 (15–99)	27.00 (15–81)	29.00 (15–87)	29.00 (16–96)	28.00 (15–99)	36.00 (15–94)	0.178
**Vinegar**								
Diarrhea	4.00 (3–18)	4.00 (3–10)	3.00 (3–18)	4.00 (3–21)	3.00 (3–21)	3.00 (3–15)	4.00 (3–21)	0.261
Dyspepsia	8.00 (4–26)	8.00 (4–20)	7.00^a^ (4–22)	8.00 (4–27)	8.00 (4–26)	9.00 (4–28)	10.00^b^ (4–28)	0.033
Constipation	5.00 (3–20)	4.00 (3–21)	4.00 (3–16)	4.50 (3–21)	4.00 (3–21)	4.00 (3–21)	4.00 (3–21)	0.469
Abdominal pain	5.00 (3–12)	5.00 (3–17)	6.00 (3–18)	5.50 (3–17)	6.00 (3–19)	7.00 (3–19)	6.00 (3–21)	0.725
Reflux	3.00 (2–11)	3.00 (2–14)	3.00 (2–14)	2.00 (2–13)	3.00 (2–13)	3.00 (2–14)	3.00 (2–14)	0.125
GIS -Total	29.00 (18–81)	25.00 (16–73)	26.00 (15–67)	28.50 (15–67)	25.00 (15–79)	29.00 (15–74)	31.00 (15–99)	0.107
**Cheese**								
Diarrhea	4.00 (3–21)	4.00 (3–21)	3.00 (3–21)	4.00 (3–21)	3.00 (3–18)	3.00 (3–10)	4.00 (3–21)	0.286
Dyspepsia	8.00 (4–28)	11.00 (4–28)	9.00 (4–28)	10.00 (4–28)	9.00 (5–23)	7.00 (4–20)	9.00 (4–27)	0.589
Constipation	4.00 (3–21)	5.00 (3–21)	5.00 (3–21)	4.00 (3–21)	4.00 (3–15)	3.00 (3–15)	3.00 (3–21)	0.074
Abdominal pain	5.00 (3–21)	7.00^a^ (3–21)	6.00 (3–19)	5.00^a^ (3–21)	7.00 (3-17)	4.50^b^ (3–8)	6.00 (3–17)	0.031
Reflux	3.00 (2–14)	3.00 (2–14)	3.00 (2–14)	3.00 (2–14)	3.00 (2–14)	2.00 (2–12)	3.00 (2–14)	0.840
GIS -Total	26.00 (15–99)	33.00 (15–94)	28.00 (15–96)	29.00 (15–99)	29.00 (20–81)	24.50 (15–52)	31.00 (15–91)	0.093
**Butter**								
Diarrhea	4.00 (3–20)	4.00 (3–21)	4.00 (3–21)	3.00 (3–21)	3.00 (3–21)	4.00 (3–20)	3.00 (3–21)	0.528
Dyspepsia	9.50 (4–28)	7.00 (4–28)	10.00 (4–28)	10.00 (4–28)	10.00 (4–23)	8.50 (4–28)	9.00 (4–28)	0.097
Constipation	4.00 (3–21)	4.00 (3–19)	4.00 (3–21)	4.00 (3–21)	4.50 (3–21)	4.00 (3–21)	6.00 (3–21)	0.498
Abdominal pain	6.00 (3–17)	5.00 (3–20)	6.00 (3–19)	6.00 (3–20)	5.00 (3–21)	6.50 (3–19)	5.00 (3–21)	0.879
Reflux	3.00 (2–14)	3.00 (2–14)	3.00 (2–14)	3.00 (2–14)	2.50 (2–14)	2.00 (2–14)	3.00 (2–14)	0.267
GIS -Total	30.00 (15–84)	25.00 (15–94)	29.00 (15–96)	30.00 (15–94)	28.00 (15–87)	29.00 (15–82)	29.00 (15–99)	0.651
**Pickle**								
Diarrhea	3.00^a^ (3–9)	3.00 (3–21)	4.00 (3–21)	4.00^b^ (3–21)	4.00 (3–21)	3.00 (3–21)	3.00 (3–21)	0.019
Dyspepsia	7.00 (4–28)	8.00 (4–28)	8.00 (4–28)	10.00 (4–28)	10.00 (4–28)	10.50 (4–28)	10.50 (4–28)	0.106
Constipation	3.00 (3–21)	4.00 (3–21)	4.00 (3–21)	5.00 (3–21)	4.00 (3–21)	4.00 (3–21)	4.00 (3–21)	0.365
Abdominal pain	5.00 (3–18)	6.00 (3–21)	6.00 (3–21)	6.00 (3–19)	6.00 (3–19)	5.00 (3–20)	5.00 (3–20)	0.679
Reflux	3.00 (2–14)	3.00 (2–14)	3.00 (2–14)	3.00 (2–14)	3.00 (2–14)	2.50 (2–14)	2.50 (2–14)	0.681
GIS -Total	23.50 (15–79)	28.50 (15–99)	28.00 (15–87)	30.00 (15–86)	30.00 (15–96)	31.00 (15–94)	31.00 (15–94)	0.405
**Sourdough bread**								
Diarrhea	4.00 (3–18)	4.00 (3–21)	3.00 (3–18)	4.00 (3–21)	3.00 (3–17)	4.00 (3–21)	4.00 (3–21)	0.720
Dyspepsia	8.00 (4–26)	7.00 (4–28)	7.00 (4–28)	10.00 (4–28)	9.00 (4–21)	8.50 (4–28)	10.00 (4–28)	0.272
Constipation	4.00 (3–20)	4.00 (3–18)	3.00 (3–20)	5.00 (3–21)	5.00 (3–21)	4.00 (3–21)	4.00 (3–21)	0.065
Abdominal pain	5.00 (3–15)	5.00 (3–20)	4.00 (3–18)	6.00 (3–19)	6.00 (3–20)	6.00 (3–18)	6.00 (3–21)	0.184
Reflux	4.00 (2–11)	3.00 (2–14)	3.00 (2–14)	3.00 (2–14)	3.00 (2–14)	3.00 (2–14)	3.00 (2–14)	0.543
GIS -Total	29.00 (15–81)	24.00 (15–94)	25.00 (15–69)	31.00 (15–96)	30.00 (15–73)	28.00 (15–91)	30.00 (15–99)	0.240
**Olive**								
Diarrhea	4.00 (3–21)	4.00 (3–21)	3.00 (3–21)	4.00 (3–20)	3.00 (3–13)	4.00 (3–21)	5.50 (3–21)	0.395
Dyspepsia	7.00 (4–28)	10.00 (4–28)	9.00 (4–28)	8.00 (4–28)	10.00 (4–24)	9.00 (4–28)	10.00 (4–28)	0.165
Constipation	4.00 (3–21)	4.00 (3–21)	5.00 (3–21)	5.00 (3–21)	4.00 (3–17)	4.00 (3–20)	4.50 (3–21)	0.600
Abdominal pain	5.00 (3–21)	6.00 (3–20)	6.00 (3–21)	6.00 (3–20)	6.00 (3–17)	6.00 (3–19)	6.00 (3–21)	0.869
Reflux	3.00 (2–14)	3.00 (2–14)	3.00 (2–14)	3.00 (2–14)	2.50 (2–14)	3.00 (2–14)	3.00 (2–14)	0.629
GIS -Total	26.50 (15–99)	30.00 (15–94)	28.00 (15–91)	28.00 (15–82)	29.50 (15–58)	30.00 (15–96)	31.50 (15–99)	0.475

**Notes.**

Data are presented as n Mean (min-max). Kruskal Wallis *H* test. *Post hoc*: Tamhane T2. GIS: Gastrointestinal System. ^a−b^: The difference between the groups shown with the same top in the same row is statistically significant (*p* < 0.05).

GIS symptoms and sub-dimensions according to the frequency of FF consumption are given in Table 3. According to the frequency of FF consumption, reflux symptoms were less common in those who consumed yogurt once in 15 days than those who consumed yogurt daily (*p* = 0.005). According to the frequency of kefir consumption, the difference in dyspepsia symptoms (*p* = 0.005) and total GIS symptoms (*p* = 0.038) was statistically significant. Accordingly, it was determined that those who consumed kefir daily experienced fewer dyspepsia symptoms and total GIS symptoms than those who consumed kefir 5–6 times a week and 1–2 times a week and less frequently. In addition, it was found that those who consumed kefir 3–4 times a week had fewer dyspepsia symptoms and total GIS symptoms compared to all groups consuming less and those consuming it 1–2 times a week, once a month and never. According to the frequency of vinegar consumption, it was determined that those consuming vinegar 3–4 times a week experienced fewer dyspepsia symptoms than those not consuming it at all (*p* = 0.033). According to the frequency of cheese consumption, it was found that those who consumed cheese once a month experienced fewer abdominal pain symptoms than those who consumed it 5–6 times a week and 1–2 times a week (*p* = 0.031). According to the frequency of pickle consumption, it was found that those who consumed pickles every day experienced fewer diarrhea symptoms than those who consumed them 1–2 times a week. This difference was statistically significant (*p* = 0.019).

To investigate the relationship between overall bowel habits and specific GI symptoms, we compared the GSRS scores among the three BSFS groups (Constipation, Normal, and Diarrhea). The results of this comparison are presented in Table 4. According to our study, reflux symptoms were seen more in constipated individuals than those with normal stool status (*p* = 0.028). In addition, the total GIS score of individuals with normal stool status in our study was significantly lower than those with constipation and diarrhea (*p* < 0.001).

**Table 4:** Comparison of gastrointestinal symptom rating scale scores across Bristol stool form scale categories.

**Symptoms**	**Stool status**	**(*n* = 546)**	**Median (min-max)**	***p***
**Diarrhea**	Constipation	82	4.00 (3–21)^a^	<0.001
	Normal	397	3.00 (3–21)^b^	
	Diarrhea	67	6.00 (3–21)^a^	
**Dyspepsia**	Constipation	82	12.00 (4–28)^a^	0.003
	Normal	397	8.00 (4–28)^b^	
	Diarrhea	67	11.00 (4–28)	
**Constipation**	Constipation	82	11.00 (3–21)^a^	<0.001
	Normal	397	4.00 (3–21)^b^	
	Diarrhea	67	4.00 (3–21)^bc^	
**Abdominal pain**	Constipation	82	6.00 (3–20)^a^	0.012
	Normal	397	6.00 (3–21)^b^	
	Diarrhea	67	6.00 (3–21)^ac^	
**Reflux**	Constipation	82	4.00 (2–14)^a^	0.028
	Normal	397	3.00 (2–14)^b^	
	Diarrhea	67	3.00 (2–14)	
**GIS total**	Constipation	82	42.00 (15–91)^a^	<0.001
	Normal	397	27.00 (15–99)^b^	
	Diarrhea	67	30.00 (15–99)^ac^	

**Notes.**

Kruskal Wallis *H* test, *Post hoc*: Tamhane T2, Median (min-max), GIS: Gastrointestinal System, BSFS: Bristol Stool Form Scale, ^a−c^: The presence of at least one common letter in the same row indicates that there is no statistically significant difference (*p* < 0.05).

The results of the sequential multiple linear regression analysis for the association between kefir consumption and total GI symptoms are presented in Table 5. In the crude model and after adjusting for demographic and lifestyle factors (Models 1–3), kefir consumption was not significantly associated with GSRS scores (*p* > 0.05). In Model 4, after additionally adjusting for the consumption of other common dairy ferments (yogurt and cheese), kefir consumption became a statistically significant predictor of lower total GI symptoms (β = 0.086, *p* = 0.048). However, this association did not remain significant when a wider range of other FF were added to the model (Models 5 and 6).

**Table 5:** Multivariate linear regression models for the effect of kefir consumption gastrointestinal symptoms.

		**Gastrointestinal symptoms**				
		β	T	95% Confidence interval		*p* value
				Lower	Upper	
**Kefir consumption**	Crude	0.081	1.895	−0.129	0.719	0.059
	Model 1	0.079	1.860	−0.194	0.709	0.063
	Model 2	0.081	1.891	−0.136	0.038	0.059
	Model 3	0.080	1.885	−0.148	0.715	0.060
	Model 4	0.086	1.984	0.037	0.744	0.048
	Model 5	0.058	1.309	−0.126	0.633	0.191
	Model 6	0.051	1.126	−0.1661	0.612	0.261

**Notes.**

Model 1: Adjusted for gender (R2: 0.018; 0.007).

Model 2: Adjusted for gender, BMI (R2: 0.019; 0.014).

Model 3: Adjusted for gender, BMI, cigarette, alcohol (R2: 0.021; 0.043).

Model 4: Adjusted for gender, BMI, cigarette, alcohol, yogurt, cheese consumption (R20.022; 0.093).

Model 5: Adjusted for gender, BMI, cigarette, alcohol, yogurt, cheese, vinegar, pickle consumption (R2: 0.036; 0.021).

Model 6: Adjusted for gender, BMI, cigarette, alcohol, yogurt, cheese vinegar, pickle, sourdough bread, ayran, butter, olive consumption (R2: 0.041; 0.045).

## Discussion

The gastrointestinal system plays an essential role in protecting and improving the individual’s health and in critical physiological functions. A disorder that may occur in this system causes many gastrointestinal symptoms, such as abdominal pain and diarrhea (Liu et al., 2023). It has been shown that approximately one in four people have gastrointestinal symptoms without any disease diagnosis (Palsson et al., 2024). It has been suggested that consumption of FFs may alleviate some gastrointestinal symptoms (Rocks et al., 2021). This study aimed to examine the effect of FF consumption on gastrointestinal symptom findings and the relationship between gastrointestinal symptoms and stool consistency in university students. The mean age of the individuals participating in our study was 21.84 ± 3.77 years, and 90.1% were female. Previous study have indicated that the prevalence of functional gastrointestinal disorders is higher in women than in men (Grosen et al., 2025; Sperber et al., 2021; Tielemans et al., 2013). When the Bristol Stool Form Scale of the students in our study was examined, it was seen that 72.7% had normal and 15% constipation type of defecation habits. Similarly, in a study conducted with university students in Japan, 13.7% of the students were found to have chronic constipation (Vu et al., 2024). Another study found that most healthy adults aged between 20–39 had normal defecation habits (Takagi et al., 2019).

FFs contain bioactive components and beneficial microorganisms that may affect gastrointestinal health (Mukherjee et al., 2024). Therefore, FFs may be potentially effective in maintaining intestinal barrier integrity, modulating microbiota, alleviating dysbiosis, and treating or preventing inflammation-related diseases (Kocot & Wróblewska, 2021). In a study conducted in Mexico, 6.8% of 311 university students consumed yogurt daily (Sousa, Baptista & Silva, 2022). A study using data from the National Adult Nutrition Survey (NANS) showed that adult individuals consumed approximately two servings (2.03 servings) of dairy products (milk, cheese, and yogurt) per day on average. It was reported that daily yogurt consumption was 26.6 ± 47.8 g (1 serving is 125 g) on average and that yogurt consumption did not exceed one serving per day (Feeney et al., 2016). According to the data from the Turkey Nutrition and Health Survey (Republic of Turkey Ministry of Health & General Directorate of Public Health, 2019) results, 51.3% of individuals consumed yogurt and Iran, and 73.9% cheese daily. This study determined that 16.1% of university students consumed yogurt, 2.4% ayran, and 32.4% cheese daily. Our results show that increasing university students’ awareness about yogurt and cheese consumption is essential for health.

Developed to assess gastrointestinal symptoms, the GSRS has been used in Turkey in patients with migraine, and individuals with diabetes and prediabetes, as well as in healthy groups as in this study (Cander et al., 2014; Karahan et al., 2020; Kaya & Turan, 2011). In our study, the participants’ mean total GSRS score was 33.66 ± 16.89. In another study conducted with university students, the majority of whom were women, the median total GSRS score was 27 (15–90) in women and 21.5 (15–71) in men (Ince Palamutoglu, Kose & Bas, 2024). In a cohort study conducted in Korea, the mean GSRS score was found to be 0.34 ± 1.01 in adult individuals without sleep disorders (Hyun, Baek & Lee, 2019). In another study conducted on young individuals with irritable bowel syndrome (IBS), the mean GSRS score was 2.6 ± .9 (Hamaguchi et al., 2020). The high score obtained from the scale by female participants indicates that women experience gastrointestinal symptoms more intensely than men.

In this study, the highest scores from the GSRS sub-factors were seen in dyspepsia, abdominal pain, and constipation. In a study conducted with healthy individuals, the majority of whom were female participants, the mean scores for the GSRS sub-factors of dyspepsia, abdominal pain, and constipation were found to be 2.0 ± 0.8, 1.6 ± 0.7, and 1.6 ± 0.8, respectively (De Graaf et al., 2022). Similar to our study, in a study conducted with Swedish adults, most individuals had a GSRS sub-factor of abdominal pain of 3 or less (Wiklund, Carlsson & Vakil, 2006). We believe that the fact that dyspepsia, abdominal pain, and constipation were the most common gastrointestinal symptoms in this study is due to the large number of female individuals.

Clinical studies have investigated the gastric and lower abdominal function of fermented dairy products. More recent randomized controlled trials have found that probiotic-enriched yogurt significantly reduces functional dyspepsia and general gastrointestinal disorders, such as bloating, epigastric pain, and postprandial fullness (Gomi et al., 2015; Yamada et al., 2024). However, the relationship between fermented dairy consumption and more specific conditions, such as reflux, appears more complex. For example, one large-scale study suggests that high dairy consumption moderates the development of reflux (Sadafi et al., 2024), while the proportions offered by this study suggest that probiotics in yogurt promote eubiosis (Herdiana, 2023). In contrast, other studies offer fragmented results. A randomized controlled trial found no significant effect of yogurt consumption on reflux interruption (Fernando et al., 2022). In addition, some cross-sectional studies have even shown that yogurt consumption, particularly in individuals with sensitivities such as acid regurgitation or lactose intolerance, is linked to the gastrointestinal monotonous pathway of purchasing (Caselli et al., 2014; Ndebia et al., 2017). Consistent with these findings, our current study found that reflux prevalence was more prevalent in individuals who consumed yogurt daily than in those who consumed it every 15 days. In conclusion, these scattered data, including our own data, strongly suggest that the relationship between yogurt consumption and reflux prevalence exists, is not fully elucidated, and may depend on individual factors.

Kefir may positively affect gastrointestinal health by modulating the composition of the intestinal microbiota and mild inflammation with the bioactive components and peptides it contains (Apalowo et al., 2024). It was observed that more than half of the individuals participating in our study (56.8%) did not consume kefir at all. However, it was found that as the frequency of kefir consumption increased, dyspepsia and total GI symptoms decreased. Our analysis of the relationship between kefir consumption and GI symptoms revealed a complex interaction. While a simple association was not statistically significant, a significant inverse association emerged after controlling for the consumption of yogurt and cheese (Model 4). This suggests a potential specific effect of kefir on GI symptoms that is independent of other common dairy ferments, possibly due to its unique and diverse microbial composition, including yeasts and specific bacterial strains not typically found in yogurt or cheese. However, it is important to note that this significant association disappeared when the model was further adjusted for a wider array of FFs (Models 5 and 6). This may indicate that while kefir has a unique profile, the overall dietary pattern of FF intake, rather than a single food item, might be more influential on GI symptoms. This complexity highlights that the simple presence or absence of one food may be less important than the broader dietary context, a finding that warrants further investigation in more controlled studies. Although it has been stated that there is almost no data on the specific benefits of vinegar for gastrointestinal symptoms (Ahuja & Ahuja, 2019), our study found that those who consumed vinegar 3–4 times a week experienced fewer dyspepsia symptoms than those who did not consume it at all.

Lactic acid bacteria found in pickled foods provide protective effects against digestive health, lowering serum cholesterol levels and infectious diseases in the gastrointestinal system (Behera et al., 2020). Our study observed that those who consumed pickles daily experienced less diarrhea than those who consumed them 1–2 times a week. No statistically significant relationship was found between the frequency of pickle consumption and other gastrointestinal symptoms. Unlike our study, a cross-sectional study conducted on individuals with functional dyspepsia showed that pickle consumption was among the foods that increased dyspepsia symptoms the most (Akhondi-Meybodi, Aghaei & Hashemian, 2015). Another study conducted on adult individuals showed that pickled food consumption was an independent risk factor for the prevalence of functional gastrointestinal disorders (Chen et al., 2024).

In a cross-sectional study, individuals with abnormal bowel health, such as constipation and diarrhea, were associated with an increased risk of developing breast cancer, colon cancer, and cardiovascular disease (Peng et al., 2022). Although the underlying mechanisms have not been fully elucidated, these findings suggest that normal bowel health may effectively prevent chronic diseases. In our study, individuals with constipation and diarrhea had more abdominal pain symptoms. According to our study, reflux symptoms were seen more in individuals with constipation. Constipation can cause increased abdominal pressure, leading to lower esophageal sphincter relaxation (Zheng et al., 2021). A study conducted in Japan showed that both gastroesophageal reflux and constipation worsened the quality of life and gastrointestinal symptoms compared to both symptoms alone. The same study found that the frequency of abnormal defecation was higher in the presence of both disorders, according to BSFS (Ogasawara et al., 2022). In a study conducted in China with high school students, constipation was observed in 11.6% of the students (Liu et al., 2023).

It is important to interpret our findings within the complex biological and commercial context of FFs. The health effects of these foods are not attributed to a single mechanism but to a combination of factors, including the activity of living microorganisms (probiotic effect), the production of microbial metabolites such as short-chain fatty acids (SCFAs) and bioactive peptides, and the transformation of the food matrix itself, which can improve digestibility (Marco et al., 2021; Mukherjee et al., 2024). Furthermore, there is a critical distinction between traditional/homemade and industrially fermented products. Many commercial products undergo post-fermentation pasteurization, which destroys live microbes and may also contain additives such as sugar or thickeners that may independently affect gastrointestinal symptoms. In contrast, traditional products generally contain higher amounts and diversity of live microorganisms (Marco et al., 2021). This variability in mechanisms and product types may explain some of the complex and nonlinear relationships observed in our study and highlights that the overall health impact of a “fermented food” is highly dependent on its specific composition and processing.

The findings of this study should be considered in light of several methodological aspects. The study’s cross-sectional design is well-suited for identifying potential associations but does not permit the establishment of a causal relationship. Furthermore, like all studies relying on dietary recall, the self-reported data may be subject to recall bias. As an observational study, it is also important to acknowledge the potential for residual confounding. While our regression models adjusted for several demographic and lifestyle factors, other variables such as overall dietary patterns and other modulators of GI health like psychological stress or physical activity levels were not assessed in this study. The interplay of these factors with FF consumption presents a valuable direction for future research. The dietary assessment was based on a food frequency questionnaire (FFQ) designed specifically for this study to capture FFs commonly consumed in Turkey. This practical approach measured consumption frequency but did not assess portion sizes, which precludes a precise dose–response analysis. Furthermore, the granular nature of the seven frequency categories resulted in small sample sizes for some of the extreme consumption groups, and these specific findings should be interpreted with caution. Future studies could build upon our findings by employing quantitative methods, such as weighed food records, and by analyzing the specific characteristics of the food products, including their live microbial content. Finally, the study population consisted of a specific demographic, primarily young female university students. While this provides important insights for this group, care should be taken when extrapolating these findings to other populations, such as men or older adults, who may have different physiological responses and dietary habits. Despite these considerations, this study provides novel, hypothesis-generating data on the relationship between the consumption of specific FFs and GI symptoms, offering a foundation for future, more controlled research in this area.

### Implications for Practice

Nutrition is a key component in the management of gastrointestinal symptoms. While the findings of our study are preliminary and do not establish causality, they highlight potential associations that may be relevant for clinical practice. For this reason, it may be beneficial for nurses and dietitians working in gastroenterology to incorporate questions about the consumption of specific FFs, such as yogurt, kefir, and pickles, into their routine dietary assessments of patients presenting with GI symptoms. Understanding a patient’s intake of these foods could provide additional context for their symptom presentation and help initiate a broader conversation about their overall dietary patterns. However, any dietary counseling should be holistic, evidence-based, and tailored to the individual, rather than being based solely on the associational findings of this study.

## Conclusions

In conclusion, it is seen that yogurt, cheese, pickles, and less consumed kefir, which are FFs commonly consumed in Turkish society, have potential health benefits in terms of reflux, dyspepsia, abdominal pain, and general gastrointestinal symptoms. Our study will contribute to the literature on the effects of FF consumption on gastrointestinal health. In addition, further studies in different populations are needed to confirm our findings and determine the association of FFs with gastrointestinal symptoms.

## Supplemental Information

10.7717/peerj.20479/supp-1 Supplemental Information 1 STROBE Checklist

10.7717/peerj.20479/supp-2 Supplemental Information 2 Dataset

10.7717/peerj.20479/supp-3 Supplemental Information 3 Fermented Foods and GI Symptoms in Students Created in BioRender.

## Additional Information and Declarations

### Data Availability

The following information was supplied regarding data availability: The data is available in the Supplemental Files.

## References

[ref-1] Ahuja A, Ahuja NK (2019). Popular remedies for esophageal symptoms: a critical appraisal. Current Gastroenterology Reports.

[ref-2] Akhondi-Meybodi M, Aghaei MA, Hashemian Z (2015). The role of diet in the management of non-ulcer dyspepsia. The Middle East Journal of Digestive Diseases.

[ref-3] Apalowo OE, Adegoye GA, Mbogori T, Kandiah J, Obuotor TM (2024). Nutritional characteristics, health impact, and applications of kefir. Foods.

[ref-4] Behera SS, El Sheikha AF, Hammami R, Kumar A (2020). Traditionally fermented pickles: how the microbial diversity associated with their nutritional and health benefits?. Journal of Functional Foods.

[ref-5] Blake MR, Raker JM, Whelan K (2016). Validity and reliability of the Bristol stool form scale in healthy adults and patients with diarrhoea-predominant irritable bowel syndrome. Alimentary Pharmacology and Therapeutics.

[ref-6] Cade J, Thompson R, Burley V, Warm D (2002). Development, validation and utilisation of food-frequency questionnaires—a review. Public Health Nutrition.

[ref-7] Cander S, Gül ÖÖ, Topyıldız F, Özkaya G, Ersoy C (2014). Comparison of prolonged release form of metformin with normal form in type 2 diabetic and prediabetic patients in terms of gastrointestinal side effects. Journal of Uludağ University Faculty of Medicine.

[ref-8] Caselli M, Zuliani G, Cassol F, Fusetti N, Zeni E, Lo Cascio N, Soavi C, Gullini S (2014). Test-based exclusion diets in gastro-esophageal reflux disease patients: a randomized controlled pilot trial. World Journal of Gastroenterology.

[ref-9] Chen C, Zhang DY, Chen S, Huang S, Zeng F, Li D, Lv YT, Xiang X, Chen RX, Zhang X, Mao F, Huang X, Wang J, Bai F (2024). Prevalence, types, and risk factors of functional gastrointestinal diseases in Hainan Province, China. Scientific Reports.

[ref-10] Chilton SN, Burton JP, Reid G (2015). Inclusion of fermented foods in food guides around the world. Nutrients.

[ref-11] Cuamatzin-García L, Rodríguez-Rugarcía P, El-Kassis EG, Galicia G, Meza-Jiménez ML, Baños Lara MDR, Zaragoza-Maldonado DS, Pérez-Armendáriz B (2022). Traditional fermented foods and beverages from around the world and their health benefits. Microorganisms.

[ref-12] De Graaf MCG, Spooren CEGM, Hendrix EMB, Hesselink MAM, Feskens EJM, Smolinska A, Keszthelyi D, Pierik MJ, Mujagic Z, Jonkers DMAE (2022). Diet quality and dietary inflammatory index in dutch inflammatory bowel disease and irritable bowel syndrome patients. Nutrients.

[ref-13] Dietrich O, Heun M, Notroff J, Schmidt K, Zarnkow M (2012). The role of cult and feasting in the emergence of Neolithic communities. New evidence from Göbekli Tepe, South-Eastern Turkey. Antiquity.

[ref-14] Dimidi E, Cox SR, Rossi M, Whelan K (2019). Fermented foods: definitions and characteristics, impact on the gut microbiota and effects on gastrointestinal health and disease. Nutrients.

[ref-15] Faul F, Erdfelder E, Lang AG, Buchner A (2007). G*Power 3: a flexible statistical power analysis program for the social, behavioral, and biomedical sciences. Behavior Research Methods.

[ref-16] Feeney EL, Nugent AP, Mc Nulty B, Walton J, Flynn A, Gibney ER (2016). An overview of the contribution of dairy and cheese intakes to nutrient intakes in the Irish diet: results from the National Adult Nutrition Survey. The British Journal of Nutrition.

[ref-17] Fernando I, Schmidt KA, Cromer G, Burhans MS, Kuzma JN, Hagman DK, Utzschneider KM, Holte S, Kraft J, Vaughan TL, Kratz M (2022). The impact of low-fat and full-fat dairy foods on symptoms of gastroesophageal reflux disease: an exploratory analysis based on a randomized controlled trial. European Journal of Nutrition.

[ref-18] García-Barón SE, Carmona-Escutia RP, Herrera-López EJ, Leyva-Trinidad DA, Gschaedler-Mathis A (2025). Consumers’ drivers of perception and preference of fermented food products and beverages: a systematic review. Foods.

[ref-19] Gomi A, Iino T, Nonaka C, Miyazaki K, Ishikawa F (2015). Health benefits of fermented milk containing Bifidobacterium bifidum YIT 10347 on gastric symptoms in adults. Journal of Dairy Science.

[ref-20] Greenwood-Van Meerveld B, Johnson AC, Grundy D (2017). Gastrointestinal physiology and function. The Handbook of Experimental Pharmacology.

[ref-21] Grosen AK, Boldsen JK, Mikkelsen S, Baunwall SMD, Dahlerup JF, Dinh KM, Bruun MT, Aagaard B, Mikkelsen C, Nissen J, Brodersen T, Petersen MS, Rostgaard K, Hjalgrim H, Sørensen E, Ostrowski SR, Pedersen OB, Hvas CL, Erikstrup C (2025). Gastrointestinal symptoms and bowel habits in 53 046 healthy Danish blood donors: a nationwide cross-sectional study. BMJ Open Gastroenterology.

[ref-22] Hall EH, Crowe SE (2011). Environmental and lifestyle influences on disorders of the large and small intestine: implications for treatment. Digestive Diseases.

[ref-23] Hamaguchi T, Tayama J, Suzuki M, Nakaya N, Takizawa H, Koizumi K, Amano Y, Kanazawa M, Fukudo S (2020). The effects of locomotor activity on gastrointestinal symptoms of irritable bowel syndrome among younger people: an observational study. PLOS ONE.

[ref-24] Herdiana Y (2023). Functional food in relation to gastroesophageal reflux disease (GERD). Nutrients.

[ref-25] Hyun MK, Baek Y, Lee S (2019). Association between digestive symptoms and sleep disturbance: a cross-sectional community-based study. BMC Gastroenterology.

[ref-26] Ince Palamutoglu M, Kose G, Bas M (2024). Probiotics and prebiotics affecting mental and gut health. Healthcare.

[ref-27] Kabak B, Dobson AD (2011). An introduction to the traditional fermented foods and beverages of Turkey. Critical Reviews in Food Science and Nutrition.

[ref-28] Karahan N, Coban O, Mete O, Toprak Celenay S (2020). Pain characteristic of gastrointestinal symptoms in individuals with migraines and its relationship to the state of disability. Anatolian Clinic Journal of Medical Sciences.

[ref-29] Kaya N, Turan N (2011). Reliability and validity of the constipation severity scale. Turkish Clinics Journal of Medical Sciences.

[ref-30] Kocot AM, Wróblewska B (2021). Fermented products and bioactive food compounds as a tool to activate autophagy and promote the maintenance of the intestinal barrier function. Trends in Food Science and Technology.

[ref-31] Kundakci S, Aktac S, Gunes FE (2021). Examination of traditional fermented food consumption and product awareness of university students in Istanbul, Turkey. North African Journal of Food and Nutrition Research.

[ref-32] Leeuwendaal NK, Stanton C, O’Toole PW, Beresford TP (2022). Fermented foods, health and the gut microbiome. Nutrients.

[ref-33] Lewis SJ, Heaton KW (1997). Stool form scale as a useful guide to intestinal transit time. Scandinavian Journal of Gastroenterology.

[ref-34] Liu T, Liu J, Wang C, Zou D, Wang C, Xu T, Ci Y, Guo X, Qi X (2023). Prevalence of gastrointestinal symptoms and their association with psychological problems in youths. Annals of Palliative Medicine.

[ref-35] Marco ML, Sanders ME, Gänzle M, Arrieta MC, Cotter PD, De Vuyst L, Hill C, Holzapfel W, Lebeer S, Merenstein D, Reid G, Wolfe BE, Hutkins R (2021). The International Scientific Association for Probiotics and Prebiotics (ISAPP) consensus statement on fermented foods. Nature Reviews. Gastroenterology & Hepatology.

[ref-36] McGovern PE, Zhang J, Tang J, Zhang Z, Hall GR, Moreau RA, Nuñez A, Butrym ED, Richards MP, Wang CS, Cheng G, Zhao Z, Wang C (2004). Fermented beverages of pre- and proto-historic China. Proceedings of the National Academy of Sciences of the United States of America.

[ref-37] Mukherjee A, Breselge S, Dimidi E, Marco ML, Cotter PD (2024). Fermented foods and gastrointestinal health: underlying mechanisms. Nature Reviews. Gastroenterology & Hepatology.

[ref-38] Narayanan SP, Anderson B, Bharucha AE (2021). Sex- and gender-related differences in common functional gastroenterologic disorders. Mayo Clinic Proceedings.

[ref-39] Ndebia EJ, Sammon AM, Umapathy E, Iputo JE (2017). Diet affects reflux in a rural African community. Acta Gastro-Enterologica Belgica.

[ref-40] Nielsen ES, Garnås E, Jensen KJ, Hansen LH, Olsen PS, Ritz C, Krych L, Nielsen DS (2018). Lacto-fermented sauerkraut improves symptoms in IBS patients independent of product pasteurisation—a pilot study. Food & Function.

[ref-41] Nkhata SG, Ayua E, Kamau EH, Shingiro JB (2018). Fermentation and germination improve nutritional value of cereals and legumes through activation of endogenous enzymes. Food Science & Nutrition.

[ref-42] Norton KI, Norton K, Eston R (2018). Standards for anthropometry assessment. Kinanthropometry and exercise physiology.

[ref-43] Ogasawara N, Funaki Y, Kasugai K, Ebi M, Tamura Y, Izawa S, Sasaki M (2022). Overlap between constipation and gastroesophageal reflux disease in Japan: results from an internet survey. Journal of Neurogastroenterology and Motility.

[ref-44] Palsson OS, Tack J, Drossman DA, Le Nevé B, Quinquis L, Hassouna R, Ruddy J, Morris CB, Sperber AD, Bangdiwala SI, Simrén M (2024). Worldwide population prevalence and impact of sub-diagnostic gastrointestinal symptoms. Alimentary Pharmacology & Therapeutics.

[ref-45] Peng Y, Liu F, Qiao Y, Wang P, Ma B, Li L, Si C, Wang X, Zhang M, Song F (2022). Association of abnormal bowel health with major chronic diseases and risk of mortality. Annals of Epidemiology.

[ref-46] Republic of Turkey Ministry of Health, General Directorate of Public Health (2019). Turkey Nutrition and Health Survey 2017 1132, Ankara. https://hsgm.saglik.gov.tr/depo/birimler/saglikli-beslenme-ve-hareketli-hayat-db/Dokumanlar/Kitaplar/Turkiye_Beslenme_ve_Saglik_Arastirmasi_TBSA_2017.pdf.

[ref-47] Revicki DA, Wood M, Wiklund I, Crawley J (1998). Reliability and validity of the gastrointestinal symptom rating scale in patients with gastroesophageal reflux disease. Quality of Life Research.

[ref-48] Rocks T, West M, Hockey M, Aslam H, Lane M, Loughman A, Jacka FN, Ruusunen A (2021). Possible use of fermented foods in rehabilitation of anorexia nervosa: the gut microbiota as a modulator. Progress in Neuro-Psychopharmacology & Biological Psychiatry.

[ref-49] Sadafi S, Azizi A, Pasdar Y, Shakiba E, Darbandi M (2024). Risk factors for gastroesophageal reflux disease: a population-based study. BMC Gastroenterology.

[ref-50] Salas-Millán JÁ, Aguayo E (2024). Fermentation for revalorisation of fruit and vegetable by-products: a sustainable approach towards minimising food loss and waste. Foods.

[ref-51] Shah AM, Tarfeen N, Mohamed H, Song Y (2023). Fermented foods: their health-promoting components and potential effects on gut microbiota. Fermentation.

[ref-52] Sousa RJM, Baptista JAB, Silva CCG (2022). Consumption of fermented dairy products is associated with lower anxiety levels in Azorean university students. Frontiers in Nutrition.

[ref-53] Sperber AD, Bangdiwala SI, Drossman DA, Ghoshal UC, Simren M, Tack J, Whitehead WE, Dumitrascu DL, Fang X, Fukudo S, Kellow J, Okeke E, Quigley EMM, Schmulson M, Whorwell P, Archampong T, Adibi P, Andresen V, Benninga MA, Bonaz B, Bor S, Fernandez LB, Choi SC, Corazziari ES, Francisconi C, Hani A, Lazebnik L, Lee YY, Mulak A, Rahman MM, Santos J, Setshedi M, Syam AF, Vanner S, Wong RK, Lopez-Colombo A, Costa V, Dickman R, Kanazawa M, Keshteli AH, Khatun R, Maleki I, Poitras P, Pratap N, Stefanyuk O, Thomson S, Zeevenhooven J, Palsson OS (2021). Worldwide prevalence and burden of functional gastrointestinal disorders, results of rome foundation global study. Gastroenterology.

[ref-54] Takagi T, Naito Y, Inoue R, Kashiwagi S, Uchiyama K, Mizushima K, Tsuchiya S, Dohi O, Yoshida N, Kamada K, Ishikawa T, Handa O, Konishi H, Okuda K, Tsujimoto Y, Ohnogi H, Itoh Y (2019). Differences in gut microbiota associated with age, sex, and stool consistency in healthy Japanese subjects. Journal of Gastroenterology.

[ref-55] Taylor BC, Lejzerowicz F, Poirel M, Shaffer JP, Jiang L, Aksenov A, Litwin N, Humphrey G, Martino C, Miller-Montgomery S, Dorrestein PC, Veiga P, Song SJ, McDonald D, Derrien M, Knight R (2020). Consumption of fermented foods is associated with systematic differences in the gut microbiome and metabolome. MSystems.

[ref-56] Tielemans MM, Jaspers Focks J, Van Rossum LG, Eikendal T, Jansen JB, Laheij RJ, Van Oijen MG (2013). Gastrointestinal symptoms are still prevalent and negatively impact health-related quality of life: a large cross-sectional population based study in The Netherlands. PLOS ONE.

[ref-57] Turan N, Ast TA, Kaya N (2017). Reliability and validity of the turkish version of the gastrointestinal symptom rating scale. Gastroenterology Nursing.

[ref-58] Turan I, Dedeli O, Bor S, Ilter T (2014). Effects of a kefir supplement on symptoms, colonic transit, and bowel satisfaction score in patients with chronic constipation: a pilot study. The Turkish Journal of Gastroenterology.

[ref-59] Valentino V, Magliulo R, Farsi D, Cotter PD, O’Sullivan O, Ercolini D, De Filippis F (2024). Fermented foods, their microbiome and its potential in boosting human health. Microbial Biotechnology.

[ref-60] Vu NTH, Quach DT, Miyauchi S, Luu MN, Yoshida M, Nguyen DTN, Yoshino A, Miyaka Y, Okamoto Y, Oka S, Hiyama T (2024). Prevalence and associated factors of chronic constipation among Japanese university students. Frontiers in Public Health.

[ref-61] Wang F, Hu D, Sun H, Yan Z, Wang Y, Wang L, Zhang T, Meng N, Zhai C, Zong Q, Hu W, Yu G, Zou Y (2023). Global, regional, and national burden of digestive diseases: findings from the global burden of disease study 2019. Frontiers in Public Health.

[ref-62] Wiklund I, Carlsson J, Vakil N (2006). Gastroesophageal reflux symptoms and well-being in a random sample of the general population of a Swedish community. American Journal of Gastroenterology.

[ref-63] World Health Organization (2004). BMI classification. https://www.who.int/data/gho/data/themes/topics/topic-details/GHO/body-mass-index?introPage=intro_3.html.

[ref-64] Yamada N, Kobayashi K, Nagira A, Toshimitsu T, Sato A, Kano H, Hojo K (2024). Beneficial effects of regular intake of Lactobacillus paragasseri OLL2716 on gastric discomfort in healthy adults: a randomized, double-blind, placebo-controlled study. Nutrients.

[ref-65] Zhang K, Bai P, Deng Z (2022). Dose-dependent effect of intake of fermented dairy foods on the risk of diabetes: results from a meta-analysis. Canadian Journal of Diabetes.

[ref-66] Zhang K, Chen X, Zhang L, Deng Z (2020). Fermented dairy foods intake and risk of cardiovascular diseases: a meta-analysis of cohort studies. Critical Reviews in Food Science and Nutrition.

[ref-67] Zhang K, Dai H, Liang W, Zhang L, Deng Z (2019). Fermented dairy foods intake and risk of cancer. International Journal of Cancer.

[ref-68] Zheng Z, Shang Y, Wang N, Liu X, Xin C, Yan X, Zhai Y, Yin J, Zhang J, Zhang Z (2021). Current advancement on the dynamic mechanism of gastroesophageal reflux disease. International Journal of Biological Sciences.

