# Supplementary figures and images for: The investigation of fermented food consumption on gastrointestinal symptoms: a cross-sectional study in university students
Source: PeerJ. 2025 Dec 16;13:e20479. doi: 10.7717/peerj.20479 (PMC12716137; doi:10.7717/peerj.20479)

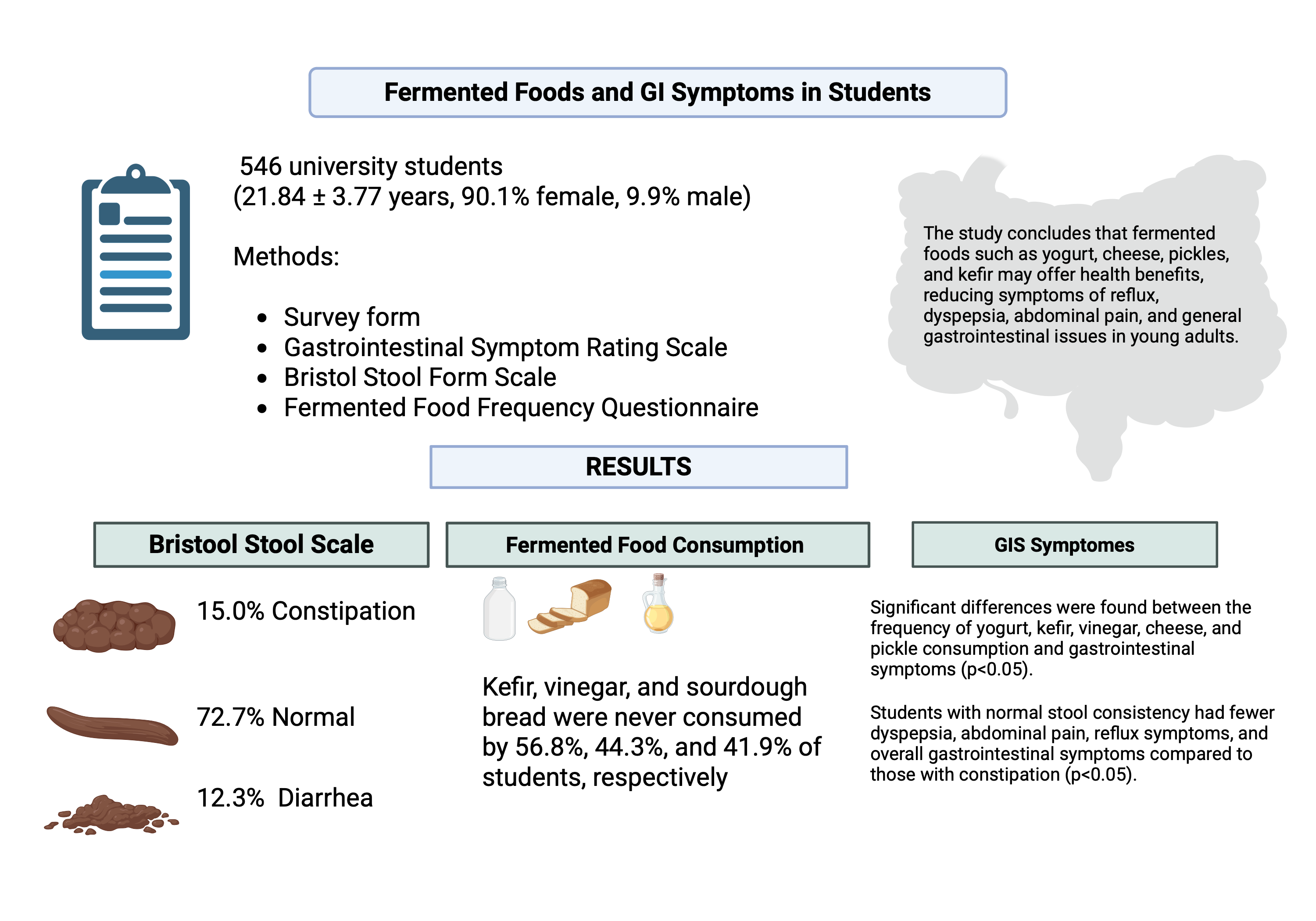

Supplement: Supplemental Information 3 — Created in BioRender. [file peerj-13-20479-s003.png]
